# Perinatal risk factors and disordered eating in children and adolescents

**DOI:** 10.1007/s40519-025-01751-2

**Published:** 2025-05-03

**Authors:** Monica Ålgars, Laura Räisänen, Sohvi Lommi, Saila Koivusalo, Heli Viljakainen

**Affiliations:** 1 Department of Psychology, Faculty of Medicine, University of Helsinki, Helsinki, Finland; 2 Faculty of Medicine and Health Technology, Tampere University, Tampere, Finland; 3 Folkhälsan Research Center, Helsinki, Finland; 4 Faculty of Medicine, University of Helsinki, Helsinki, Finland; 5 Department of Obstetrics and Gynecology, HUS Helsinki University Hospital, Helsinki, Finland

**Keywords:** Disordered eating, Eating disorders, Perinatal factors, Pregnancy, Childbirth

## Abstract

**Objective:**

Studies have reported associations between perinatal factors (obstetric and neonatal factors) and later eating disorder risk. However, previous findings have been partly conflicting. Here, we analyzed associations between perinatal factors and disordered eating in a large cohort of Finnish children and adolescents.

**Method:**

The participants were 8- to 14-year-old children and adolescents (*N* = 11,357) from The Finnish Health in Teens study. Disordered eating was assessed using the Children’s Eating Attitudes Test (ChEAT). Perinatal data were obtained from the Finnish Birth Registry. Perinatal variables were initially analyzed using Chi-square analyses and linear regressions. Variables associated with disordered eating (*p* < .10) were entered into a multinomial logistic regression model. The regression analysis was conducted both including and excluding maternal BMI, as this information was missing for > 80% of the participants.

**Results:**

Of the participants, 56.6% reported disordered eating (ChEAT score ≥ 11) or partial disordered eating (1–10) symptoms. Including maternal BMI in the analyses (*n* = 1921), higher levels of disordered eating were independently associated with maternal pre-pregnancy BMI (OR 1.07, 95% CI [1.02, 1.12]), maternal smoking during pregnancy (OR 2.64, 95% CI [1.49, 4.68]), urgent or emergency cesarean birth (OR 2.16, 95% CI [1.10, 4.05]). Assisted reproduction was associated with lower levels of disordered eating (OR 0.39, 95% CI [0.20, 0.76]).

**Discussion:**

The results suggest that pregnancy and childbirth are vulnerable developmental periods, associated with later eating pathology. Further studies disentangling genetic and environmental mechanisms of associations between perinatal factors and later eating pathology are needed.

**Level of evidence:**

Level III, Evidence obtained from well-designed cohort or case–control analytic studies.

**Supplementary Information:**

The online version contains supplementary material available at 10.1007/s40519-025-01751-2.

## Introduction

Eating disorders are serious psychiatric disorders characterized by abnormal eating or weight-control behaviors[1]. Disordered eating refers to eating attitudes and behaviors such as drive for thinness, dieting, body dissatisfaction, and binge eating. Disordered eating does not necessarily meet diagnostic criteria for eating disorders, but may nonetheless have serious psychological and physiological consequences[2]. Disordered eating in children and adolescents has been associated with, e.g., obesity, depression, greater substance use, suicide ideation or attempts, lower health-related quality of life, as well as later eating disorders [3–7]. Among children and adolescents, disordered eating is more common than diagnosable eating disorders and has been described as a public health challenge [3, 8].

Research suggests that pregnancy, birth, and early life are vulnerable developmental periods that may affect later psychiatric risk, including eating disorder risk. For instance, perinatal factors (i.e., obstetric factors and neonatal factors) have been associated with feeding and eating disorders in small children (0–3 years) as well as in adults [9–14]. Previously studied perinatal factors that in some studies have been associated with later eating disorder risk include higher maternal age, maternal smoking during pregnancy, gestational diabetes, preeclampsia, very preterm birth (< 32 weeks), hypoxic complications at birth, lower birth weight, and being the firstborn child [9, 11, 12, 14]. However, the results have partly conflicted across studies, and not all findings have been replicated. Conflicting findings include associations between eating disorders and maternal age, gestational diabetes, maternal smoking, obstetric complications, cesarean section delivery, Apgar score, and gestational age [11, 14]. Previous studies have been based on samples of various sizes (ranging from < 100 to several thousand participants), and some have been limited by poor representativeness [14]. Adequately powered replication studies, such as the present study, have thus been called for [11, 14]. To our knowledge, no previous studies on the above-mentioned perinatal factors have focused on the risk for later disordered eating, only diagnosable eating disorders. 

In the present study, perinatal factors and their association with later disordered eating were studied in a large population of Finnish children and adolescents. We hypothesized that maternal adverse health symptoms and smoking during pregnancy, obstetric complications, and neonatal adverse health symptoms would be associated with higher levels of disordered eating during late childhood and early adolescence.

## Methods

### Participants

This study was an observational retrospective study based on the Finnish Health in Teens (Fin-HIT) prospective cohort, established to study the well-being of Finnish children and adolescents across Finland. Participants (*n* = 11,407) were recruited in 2011–2014 during school visits where they filled in a questionnaire regarding health aspects, including disordered eating. In addition, data on perinatal factors were obtained from a nationwide register. More detailed characteristics of this cohort have been described elsewhere [15]. Of the study population, 5981 (52.4%) were girls, and 5423 (47.6%) were boys (information about gender missing *n* = 3).

### Measures

#### Disordered eating

Disordered eating was assessed using the Children’s Eating Attitudes Test (ChEAT) [16]. ChEAT is a 26-item questionnaire assessing eating attitudes and disordered eating behaviors in children aged 8–15. The items are scored on a 6-point Likert scale, with answer options ‘never’, ‘rarely’, ‘sometimes’, ‘often’, ‘usually’, and ‘always’. A factor analysis of the ChEAT has previously been conducted in a Finnish sample, resulting in four factors: concerns about weight, limiting food intake, pressure to eat, and concerns about food [17]. The psychometric qualities of the ChEAT have been validated in Finnish children and adolescents aged 10–15 years [17]. A sum variable (composite score) was computed for the 24-item ChEAT [17] for the present analyses. Cronbach’s alpha for the sum variable was 0.82, indicating good internal consistency.

For the analyses, the participants were divided into three groups based on their ChEAT score: 0 (no disordered eating symptoms), 1–10 (partial disordered eating symptoms), and ≥ 11 (disordered eating symptoms). There is no consensus on the appropriate cut-off for the ChEAT score; previous studies have examined cut-offs ranging from 10 to 20 [18]. Instead of using a dichotomous variable, we decided to analyze participants that reported some disordered eating symptoms as a separate group, because only a small part (5.4%) of the participants reported scores above a cut-off of 10. Data for the ChEAT score were missing for 803 participants (7%), resulting in their exclusion from the present study and a final sample size of 11,357.

#### Perinatal data

Perinatal data were obtained from the Finnish Medical Birth Register and linked with the Fin-HIT questionnaire data using personalized social security numbers, unique to each Finnish resident. The Finnish Medical Birth Register contains data on all mothers who have given birth in Finland and on all newborns until the age of 7 days [19].

Based on available data, previous research on the topic, and clinical evaluation, the following perinatal variables were included in the study. Maternal variables: maternal age; maternal BMI pre-pregnancy; maternal smoking during pregnancy; assisted reproduction (in vitro fertilization, intracytoplasmic sperm injection, or frozen embryo transfer); thromboprophylaxis; insulin treatment started during pregnancy; hospitalization during pregnancy due to preeclampsia. Neonatal variables: being the firstborn child or not; very preterm birth (< 32 weeks); mode of delivery; asphyxia; birth weight; birth length; 1-min Apgar score; care in an intensive care unit or an observational ward after delivery; respirator care; resuscitation; and antibiotic treatment.

### Data analyses

The association between each perinatal variable and the three-level ChEAT score was initially analyzed using crosstabs and Chi-square analyses for categorical perinatal variables and linear regression for continuous perinatal variables. Perinatal variables for which the association had a *p*-value > 0.10 were excluded from further analyses. A multinomial logistic regression model was used to analyze the association between all remaining perinatal variables and levels of disordered eating. The reference category was “No disordered eating”. Information about the mother’s pre-pregnancy BMI was missing for 82.3% of the participants. Therefore, the multinomial logistic regression analysis was conducted both including and excluding maternal BMI. All data analyses were performed using IBM SPSS Statistics 28.0 and a 2-sided *p*-value of < 0.05 was considered statistically significant for the regression model.

## Results

### Descriptive statistics

The age of the participants ranged from 8.33 to 14.33 (*M* = 11.17, *SD* = 0.84) years. A linear regression analysis showed no significant association between the participants’ age and levels of disordered eating (*F* = 0.68, *p* = 0.79). The mother’s age at the time of the participant’s birth ranged from 13.95 to 45.97 years (*M* = 30.34, *SD* = 5.19). Of the 11,357 participants, 43.4% had a ChEAT score of 0 (no disordered eating symptoms); 51.2% had a ChEAT score of 1–10 (partial disordered eating symptoms); and 5.4% had a ChEAT score ≥ 11 (disordered eating symptoms). Maternal and perinatal factors, across levels of disordered eating, are outlined in more detail in Table 1.

**Table 1:** Descriptive statistics of perinatal factors across disordered eating categories (*n* = 11,357). Results from linear regression analyses and Chi-square analyses

	No disordered eating (ChEAT score = 0)	Partial disordered eating symptoms (ChEAT score 1–10)	Disordered eating symptoms (ChEAT score ≥ 11)	*F*	Missing data (%)
Maternal age (*M, SD*)	30.17 (5.11)	30.35 (5.23)	29.73 (5.39)	0.01	4.1
Maternal BMI (kg/m^2^) pre-pregnancy (*M, SD*)	23.28 (3.86)	23.44 (4.03)	24.60 (4.90)	6.62**	82.3
Birth weight (kg) (*M, SD*)	3.35 (0.55)	3.50 (0.57)	3.49 (0.51)	3.78*	4.8
Birth height (cm) (*M, SD*)	50.19 (2.45)	50.14 (2.49)	49.94 (2.23)	3.65*	4.8
				*χ*2	
Assisted reproduction (in vitro fertilization, intracytoplasmic sperm injection, or frozen embryo transfer) (*n*, valid %)	26 (0.6)	16 (0.3)	1 (0.2)	4.98*	4.4
Firstborn child (*n*, valid %)	2108 (47.4)	2270 (44.1)	211 (40.3)	15.81***	4.5
Insulin treatment started during pregnancy (*n*, valid %)	5 (0.1)	7 (0.1)	3 (0.6)	6.85*	4.4
Hospitalization during pregnancy due to preeclampsia (*n*, valid %)	189 (4.2)	222 (4.3)	25 (4.8)	0.32	4.4
Prematurity (< 32 weeks) (*n*, valid %)	33 (0.7)	39 (0.8)	1 (0.2)	2.17	4.6
Asphyxia (*n*, valid %)	118 (2.7)	121 (2.3)	18 (3.4)	2.77	4.4
Care in an intensive care ward or an observation ward after delivery (*n*, valid %)	448 (10.1)	517 (10.0)	50 (9.6)	0.16	4.4
Respirator care (*n*, valid %)	56 (1.3)	62 (1.2)	8 (1.5)	0.44	4.4
Resuscitation (*n*, valid %)	25 (0.6)	35 (0.7)	2 (0.4)	1.0	4.4
Antibiotic treatment (*n*, valid %)	211 (4.7)	233 (4.5)	24 (4.6)	0.30	4.4
Mode of delivery (*n*, valid %)				8.56*	4.5
Vaginal delivery	3739 (84.1)	4240 (82.2)	430 (82.4)		
Elective cesarean birth	339 (7.6)	433 (8.4)	37 (7.1)		
Urgent or emergency cesarean birth	368 (8.3)	488 (9.5)	55 (10.5)		
1-min Apgar score (*n*, valid %)				2.94	4.5
0–3 (low)	49 (1.1)	49 (0.9)	3 (0.6)		
4–6 (moderately abnormal)	148 (3.3)	193 (3.7)	21 (4.0)		
7–10 (reassuring)	4248 (95.6)	4923 (95.3)	499 (95.4)		
Maternal smoking during pregnancy (*n*, valid %)				36.83***	7.1
No smoking	3959 (91.4)	4514 (90.0)	421 (83.2)		
Quit smoking during the 1 trimester	72 (1.7)	91 (1.8)	13 (2.6)		
Smoking after the 1 trimester	300 (6.9)	413 (8.2)	72 (14.2)		

*p < 0.10, **p < 0.01, ***p < 0.001

F values are based on linear regression analyses, *χ*2 are based on Chi-square analyses

### Perinatal factors and disordered eating

**Fig. 1:**
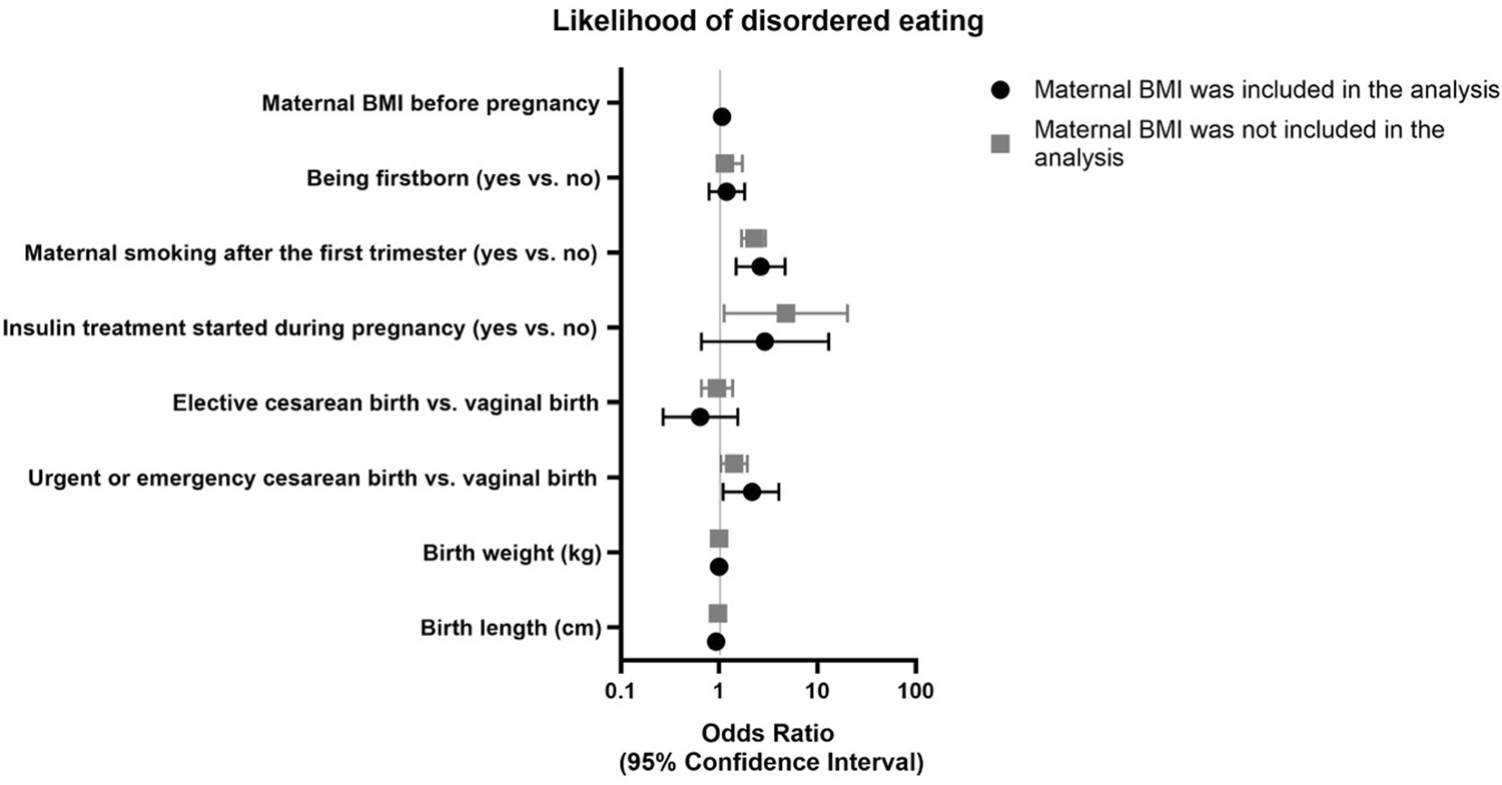
Associations between perinatal factors and later disordered eating. Results from a multinomial logistic regression analysis

When maternal pre-pregnancy BMI was included in the multinomial logistic regression model (*n* = 1921), higher maternal pre-pregnancy BMI (OR 1.07, 95% CI [1.02, 1.12]), smoking after the first trimester (OR 2.64, 95% CI [1.49, 4.68]), and urgent or emergency cesarean birth (OR 2.16, 95% CI [1.10, 4.05]) predicted an increased likelihood of having disordered eating compared to no disordered eating. Assisted reproduction predicted a decreased likelihood of having partial disordered eating symptoms compared to no disordered eating symptoms (OR 0.39, 95% CI [0.20, 0.76]). The results are outlined in Fig. 1 and supplementary Table 2.

Excluding maternal pre-pregnancy BMI from the multinomial logistic regression model (*n* = 9189) and thus not controlling for maternal BMI, smoking after the first trimester (OR 2.28, 95% CI [1.69, 2.95]), insulin treatment started during pregnancy (OR 4.75, 95% CI [1.12, 20.15]), and urgent or emergency cesarean birth (OR 1.42, 95% CI [1.04, 1.94]) predicted an increased likelihood for disordered eating compared with no disordered eating. Assisted reproduction (OR 0.44, 95% CI [0.23, 0.85]) and being the firstborn child (OR 1.14, 95% CI [1.16, 1.72]) predicted a decreased likelihood of disordered eating. The results are described in Fig. 1 and supplementary Table 3.

## Discussion

In this observational retrospective study using a large cohort of Finnish children and adolescents, associations between perinatal risk factors and later disordered eating were investigated. Higher levels of disordered eating were significantly and independently associated with maternal pre-pregnancy BMI, maternal smoking during pregnancy, and urgent or emergency cesarean birth. Lower levels of disordered eating were associated with assisted reproduction. Excluding maternal BMI from the analyses and thus not controlling for it, insulin treatment during pregnancy additionally increased, and being the firstborn child additionally reduced, the likelihood of disordered eating.

Our findings confirmed previous results regarding maternal smoking during pregnancy and urgent or emergency cesarean birth [14]. However, it should be noted that not all studies have found an association between these perinatal factors and later eating pathology risk [14]. In contrast with previous findings on clinical eating disorders [9, 11, 12, 14], preeclampsia, gestational diabetes, very preterm birth (< 32 weeks), higher maternal age, obstetric complications such as asphyxia, birth weight, and being the firstborn child did not predict higher levels of later disordered eating in our study. Neonatal factors such as antibiotic treatment, resuscitation, 1-min Apgar score, or care in an intensive care unit or observational unit after delivery were likewise unrelated to later disordered eating in this study.

Maternal smoking during pregnancy has previously been associated with a later risk for psychiatric disorders, even when controlling for genetic and familial factors [20]. Prenatal smoking exposure has been described to alter fetal brain development as well as brain structure and function in childhood [20]. It is therefore plausible that neurodevelopmental factors partly explain the present detected association between maternal smoking during pregnancy and later disordered eating.

Previously, associations between cesarean section birth and later obesity [21] as well as neurodevelopmental disorders [22] have been reported. However, it has been suggested that these associations are mostly explained by confounding factors such as maternal BMI or familial factors [21, 22]. We can likewise not conclude whether the associations detected in this study are causal. Although we did control for maternal BMI, the present associations between perinatal factors and later levels of disordered eating may at least partly be explained by other confounding factors. To disentangle environmental and potential genetic mechanisms of these detected associations, genetically informed studies on the topic have been called for [9, 11].

Interestingly, assisted reproduction predicted a smaller likelihood of later disordered eating compared to no disordered eating symptoms. Parents who seek and receive assisted reproduction may differ from the general population regarding aspects that may also affect later psychiatric risk, i.e., this detected association may be explained by factors not controlled for here.

## Strengths and limitations

Key strengths of the study included a large sample size, a longitudinal design, and the inclusion of perinatal factors that have not previously been largely studied. Most previous studies have focused on diagnosable eating disorders, while the present study included data on more broadly defined disordered eating. We also included perinatal factors that have not previously been largely studied (such as assisted reproduction, hospital care after birth, and antibiotic treatment), thus adding novel knowledge to the body of evidence. A main limitation of the study was the substantial amount of missing data for maternal BMI before pregnancy. It should also be noted that the participation rate for the Fin-HIT study was 36%, which may limit the generalizability of the results. An additional limitation is that no measures of maternal stress were included in the study, despite previous research having found associations between maternal stress during pregnancy and later eating disorders in their children [14].

## Conclusions

In sum, our study adds to the literature highlighting the complex etiology of eating pathology. It has been suggested that pregnancy and early life are vulnerable developmental periods that may impact neurodevelopment and epigenetic changes, and thus affect vulnerability to eating pathology later in life. From a clinical standpoint, the present study adds to the body of literature suggesting that maternal smoking during pregnancy predicts later eating pathology risk and is clearly advised against. A nuanced perception of the etiology of eating pathology also aids clinicians and patients in understanding that disordered eating is not a personal choice, or a merely psychological or cultural phenomenon, but also may encompass biological risk factors. 

## What is already known on this subject?

- Perinatal factors (i.e., obstetric and neonatal factors) are associated with later feeding and eating disorders in children and adults.
- Such factors include higher maternal age, maternal smoking during pregnancy, gestational diabetes, preeclampsia, very preterm birth (< 32 weeks), hypoxic complications at birth, lower birth weight, and being the firstborn child.
- However, previous results have been partly conflicting.

## What this study adds?

- This study focused on disordered eating instead of clinical eating disorders.
- In children and adolescents, maternal pre-pregnancy BMI, maternal smoking during pregnancy, and urgent or emergency cesarean birth are associated with higher levels of disordered eating. Assisted reproduction is associated with lower levels of disordered eating.
- These results support a nuanced understanding of the etiology of eating pathology, also encompassing biological risk factors.

## Supplementary Information

Below is the link to the electronic supplementary material.

**Supplementary Material 1. **Supplementary Table 2. Associations of perinatal factors with later disordered eating, including maternal BMI (*n* = 1921). Results from a multinomial logistic regression analysis. Supplementary Table 3. Associations of perinatal factors with later disordered eating, not including maternal BMI (*n* = 9819). Results from a multinomial logistic regression analysis.

## Data Availability

The dataset generated during the current study is not publicly available due to privacy restrictions. Anonymized data that support the findings of this study are available upon reasonable request from the corresponding author.
