# Supplementary material for: Perinatal risk factors and disordered eating in children and adolescents
Source: Eat Weight Disord. 2025 May 3;30(1):40. doi: 10.1007/s40519-025-01751-2 (PMC12049285; doi:10.1007/s40519-025-01751-2)
Supplement: Supplementary file 1 — Supplementary Material 1. Supplementary Table 2. Associations of perinatal factors with later disordered eating, including maternal BMI (n = 1921). Results from a multinomial logistic regression analysis. Supplementary Table 3. Associations of perinatal factors with later disordered eating, not including maternal BMI (n = 9819). Results from a multinomial logistic regression analysis. [file 40519_2025_1751_MOESM1_ESM.docx]

**Supplementary Table 2.** Associations of perinatal factors with later disordered eating, including maternal BMI (*n* = 1921). Results from a multinomial logistic regression analysis

|  | Dependent variable category^a^ | | | |
| --- | --- | --- | --- | --- |
| Perinatal factors | Partial disordered eating symptoms | | Disordered eating symptoms | |
|  | Exp(B) | 95% CI | Exp(B) | 95% CI |
| Maternal BMI before pregnancy | 1.01 | [.99, 1.04] | **1.07**** | [1.02, 1.12] |
| Assisted reproduction |  |  |  |  |
| No^b^ |  |  |  |  |
| Yes | **0.39**** | [0.20, 0.76] | n/a | n/a |
| Being the firstborn child |  |  |  |  |
| No^b^ |  |  |  |  |
| Yes | 1.17 | [0.96, 1.41] | 1.19 | [0.79, 1.81] |
| Maternal smoking during pregnancy |  |  |  |  |
| No smoking^b^ |  |  |  |  |
| Quit smoking during the 1 trimester | 1.19 | [0.60, 2.37] | 2.37 | [0.80, 7.07] |
| Smoking after the 1 trimester | 1.04 | [0.73, 1.48] | **2.64**** | [1.49, 4.68] |
| Insulin treatment started during pregnancy |  |  |  |  |
| No^b^ |  |  |  |  |
| Yes | 1.09 | [0.34, 3.49] | 2.92 | [0.66, 13.00] |
| Mode of delivery |  |  |  |  |
| Vaginal birth^b^ |  |  |  |  |
| Elective caesarean birth | 1.29 | [0.92, 1.83] | 0.64 | [0.27, 1.55] |
| Urgent or emergency caesarean birth | **1.56*** | [1.08, 2.26] | **2.16*** | [1.10, 4.05] |
| Birth weight | 1.00 | [1.00, 1.00] | 1.00 | [1.00, 1.00] |
| Birth length | 1.02 | [.94, 1.09] | 0.93 | [0.80, 1.08] |

*Notes.*

^a^Dependent variable category: “No disordered eating symptoms“ is the reference category.

^b^Reference categories

**p* < .05, **p < .01

n/a There were too few cases to perform the analysis.

**Supplementary Table 3**. Associations of perinatal factors with later disordered eating, not including maternal BMI (*n* = 9819). Results from a multinomial logistic regression analysis

|  | Dependent variable category^a^ | | | |
| --- | --- | --- | --- | --- |
| Perinatal factors | Partial disordered eating symptoms | | Disordered eating symptoms | |
|  | Exp(B) | 95% CI | Exp(B) | 95% CI |
| Assisted reproduction |  |  |  |  |
| No^b^ |  |  |  |  |
| Yes | **0.44*** | [0.23, 0.85] | n/a | n/a |
| Being the firstborn child |  |  |  |  |
| No^b^ |  |  |  |  |
| Yes | **1.18**** | [1.09, 1.29] | **1.14**** | [1.16, 1.72] |
| Maternal smoking during pregnancy |  |  |  |  |
| No smoking^b^ |  |  |  |  |
| Quit smoking during the 1 trimester | 1.14 | [0.83, 1.16] | 1.76 | [0.96, 3.21] |
| Smoking after the 1 trimester | **1.21*** | [1.03, 1.41] | **2.28**** | (1.69, 2.95] |
| Insulin treatment started during pregnancy |  |  |  |  |
| No^b^ |  |  |  |  |
| Yes | 1.89 | [0.38, 3.75] | **4.75*** | [1.12, 20.15] |
| Mode of delivery |  |  |  |  |
| Vaginal birth^b^ |  |  |  |  |
| Elective caesarean birth | 1.15 | [0.00 1.14] | 0.95 | (0.66, 1.37] |
| Urgent or emergency caesarean birth | **1.21*** | [1.04, 1.40] | **1.42*** | [1.04, 1.94] |
| Birth weight | 1.00 | [1.00, 1.00] | 1.00 | [1.00, 1.00] |
| Birth length | 1.02 | [0.99, 1.05] | 0.97 | [0.91, 1.04] |

*Notes.*

^a^Dependent variable category: “No disordered eating symptoms“ is the reference category.

^b^Reference categories

**p* < .05, **p < .01

n/a There were too few cases to perform the analysis.
